# Urgency to rein in the gene-editing technology

**DOI:** 10.1007/s13238-015-0161-5

**Published:** 2015-04-28

**Authors:** Xiaoxue Zhang

In the past few years, we have witnessed and been excited by biotechnological advances that allow for the precise editing of genomic information in living eukaryotic cells. Facilitated by TALEN (transcription activator-like effector nucleases) or CRISPR (clustered regularly interspaced short palindromic repeats) systems, scientists can now routinely perform gene-editing in a variety of organisms including plants, insects, rodents and primates, as well as in human somatic cells in basic research. These innovative techniques have opened up new opportunities for scientific discovery and human disease therapeutic development that were not possible just a few years ago. Recently, scientists try to apply the gene-editing techniques to human germ cells and embryos, hoping to prevent the transmission of inherited diseases to the next generation in the future. However, these rapid advances in the capability of genomic manipulation have begun to outpace the scope of existing regulatory agencies and laws. As recently attested by prominent scientists in the field, in light of safety and ethical concerns associated with potential applications of these gene-editing techniques, it is time for the scientific community, public, funding bodies and governments to come together to re-define and reinforce the boundaries of gene-editing research.

With extraordinary care, consideration and deliberation, *Protein & Cell* has decided to publish in this issue a scientific study that reports CRISPR-based gene-editing of human tripronuclear zygotes (fertilized zygotes with one oocyte nucleus and two sperm nuclei). Utilizing tripronuclear zygotes, which occur in common *in vitro* fertilization (IVF) procedures and are clinically discarded because they are unable to develop at later stages, the authors demonstrate that CRISPR-based gene-editing can be achieved in this setting. However, the authors also report notable off-target effects of CRISPR-based gene-editing, low efficiency of homologous recombination directed repair (HDR), mosacism and unwanted mutations, substantiating the concerns that the therapeutic application of these new techniques could have unpredictable safety risks. Because germline modification is permanent and heritable, it should be given the particular concerns. *Protein & Cell* has fully realized that this study can provide direct evidences to address some of the safety concerns of the CRISPR/Cas9 technique. It may also raise a series of questions and bring further controversies to the field of gene-editing research. In this unusual situation, the editorial decision to publish this study should not be viewed as an endorsement of this practice nor an encouragement of similar attempts, but rather the sounding of an alarm to draw immediate attention to the urgent need to rein in applications of gene-editing technologies, especially in the human germ cells or embryos.

Biomedical research is not unfamiliar with such controversies; in fact, many landmark breakthroughs have been met with safety, legal and ethical questions. This was the case for the IVF procedure and recombinant DNA techniques in the 1970s, the mammalian cloning technique (i.e., the birth of the Dolly sheep) in the 1990s, the generation of inducible pluripotent stem cells (iPSC) in the 2000s and mitochondrial DNA (mtDNA) transfer in embryos in 2013, to name just a few in recent memory. Those past scientific and public debates should not only serve as successful precedents that the dialogue on gene-editing technologies will follow, but also offer us the courage, confidence and wisdom to solve the challenges we are currently facing. By working together with both the public and governments, the research community should immediately and comprehensively assess the benefits and risks associated with any potential application of gene-editing techniques. Until a consensus on new regulatory rules can be reached, it is in the best interest of all parties that the research field should voluntarily avoid any study that may pose potential safety and/or ethical risks. Only by holding themselves to the highest standards will scientists retain the public’s trust in biomedical research, and at the same time, provide the best service for the well-being of our society.

